# Systemic Medication in Dental Practice

**Published:** 1894-03

**Authors:** Edgar Palmer

**Affiliations:** La Crosse, Wis.


### Systemic Medication in Dental Practice.

BY EDGAR PALMER, D.D.S., LA CROSSE, WIS.

Read in the Section on Dental and Oral Surgery, at the Forty-fourth Annual Meeting of the American Medical Association.

Let us consider first our specialty in its physiologic and pathologic aspect.

It is supposed that all who are associated with this Section are of the opinion that "dentistry is a specialty of medicine."

It is sufficient for me to base my remarks upon the simple fact that an all-wise Creator has given man organs of mastication much after the same manner and for an equally important service as the organs of hearing and sight, and these organs being subject to abuse, disease, and functional derangements, thousands of persons are required to devote their lives to their care and treatment. I am aware it is asserted that this great army of scientific men are, by the mechanical character of their methods, too far removed from the province of general medicine to be entitled to the privileges accorded to the oculist or aurist in giving attention to functional disturbances remote from organs over which they have special charge. I confess that my eyes are too weak to see the distinction, and my ears too dull to hear the voice of duty calling louder in one case than another. All the ambition and desire of the last years of my school life was centered upon the one thought of some day entering upon the practice of general medicine and surgery. An empty purse obliged me to take up something else, and as a temporary expedient I took my place at the foot of the ladder upon which the dental profession was then slowly climbing toward the distinguished eminence which it now occupies in the department of science.

The status of dentistry to-day is most gratifying. For many years every force and pressure of intellectual, progressive effort has been brought to bear upon the creation of a higher standard of qualification; and while we have done much, there is opportunity to do more to exalt and commend our services in this general and beneficent cause of humanity.

I can confidently say that now, after more than thirty years' constant practice of this specialty, I am as near the practice of general medicine as I ever expect to be. This branch of the healing art, with its achievements, is good enough, and its resources and beneficent possibilities stimulating enough for any purpose save avarice and greed. The words of Daniel Webster in relation to the practice of law are so germane to this thought that I quote them here:

"Our profession is good if practiced in the spirit of it; it is damnable fraud and iniquity when its true spirit is supplied by a spirit of mischief-making and money-getting. The love of fame is extinguished; every ardent wish for knowledge repressed; conscience put in jeopardy; and the best feelings of the heart indurated by the mean, money-catching, abominable practices which cover with disgrace some of the modern practitioners of law."

The application of the established principles of general medicine was a matter but little considered in the first years of my practice. I frankly say it is comparatively but little understood by me to-day. This much we do know, however, that it is the province of the dentist to search for bodily manifestations in health and disease which may bear upon the interpretation of dental function. We do not deal with the human teeth as though they were a mere mechanism of bone and pulp and nerve, or deal with their functions as an independent agent, with little or no concern or relation to other organs and the whole life of the being, but recognize a vital harmony and essential interdependence—the orderly subordination and co-ordination of parts each dependent upon the other for functional activity.

The magnetic influence and electric force of all nerve-supply, and the effects of fully charged abundant blood courses, bring to our aid a health-giving action upon which we can build pledges of success, while, on the other hand, lacking in vitality, a system with evidences of derangement in formidable disturbances, neurasthenic in character, we are safe in predicting an aggravation if not the direct source of dental lesions.

The only conspicuous inheritance three-fourths of the human race confer upon their offspring is a stunted development and impaired structural formation of the different organs that so universally beget disease.

Temperament and disposition are not more certainly marked upon the child than the impoverished condition of all nerve and blood-supply, from defective nutrition and assimilation, and as such conditions in the child are rarely obliterated during life, they should not be neglected in our practice for physiologic, pathologic, and diagnostic purposes at any time.

The organic origin of pathologic processes influencing local lesions, has for many years impressed itself upon my mind as a subject worthy of our earnest consideration. As long ago as 1878 I was permitted to read a paper before the American Dental Association upon this subject. I say "permitted," because that honorable body has a peculiar system of quarantine, requiring every immigrant, suspected of having contracted germs of progress, to be searched. My paper when returned to me bore a verbal indorsement something like this, in sentiment: We, the undersigned Sanitary Committee, have discovered in this, microscopic traces of an idea, but as we do not regard it of sufficient virility to infect any great number, you have our permission to read it. Whether the idea discovered by that Committee was what I intended to embody in my effort or not, I never learned; but if so, it was this: That the hyperemic condition consequent upon the saturation of the tissues of the body with alcohol, so strangulated the vascular and nerve-supply as to cut off proper nourishment from the dental membranes, and indirectly establishing retrograde metamorphosis of the nerve-fibrils of the tooth, prolific also of neuralgia, and often attended by wasting of tissue, as in decay, under such circumstances of impaired nutrition.

The mysterious force which inhibits itself in the sensitive protoplasm of a muscle is a phenomenon of no less interest to us than to the general practitioner.

The scientific investigations carried on by dentists during the last decade, to put this profession, as well as medical men, in possession of agents which lessen the vital activity of pathogenic parasites, is evidence of an intellectual and progressive spirit most commendable, as well as interesting and valuable to all practitioners of the healing art.

Why, then, let me ask, in view of all that our profession has achieved in this line of scientific work, and in view of our rigid system of dental education in all branches which general medicine requires of other specialists, are we prohibited from exercising this knowledge we are expected to possess, in the treatment of such systemic conditions as directly influence a dental lesion?

Brought daily face to face with all these exciting and retarding influences which you so well understand, must we forever lock the door leading out of the oral vestibule into the wonderful habitation of man's vital forces, and turn the key over to the family physician?

How many dentists, out of two hundred in this country of whom I recently asked the question, do you suppose answered that they prescribed constitutional treatment independent of the counsel or advice of the physician? The number is too few to stimulate me in carrying on the investigation any further.

But notwithstanding the majority of our profession, men whose views are worthy of the highest regard, express themselves as adverse to the practice of administering medicines themselves, I believe the day will come when it shall be considered within the province of the medically educated dentist to make recommendations for general physical ailments affecting the organs given into his care. There must always be a courteous recognition of the rights and duties of the physician, but *within a common sense limit.* I believe "it is better to experience the truth and enjoy its practical uses, even without a perfect knowledge of every theory, than to have full mastery of the theory, but reap no benefits from it."

Dr. J. Taft said that it was in the experience of every practitioner of dentistry to have the question raised about the need of systemic medication as a necessary part of the treatment of diseases of the mouth and teeth. He could see no reason why a dentist should not modify by treatment the systemic conditions which hindered the success of his operations in the special line of the profession which he practiced. Why should he not treat the system, as well as the oculist or any other specialist who found the patient in an unfit condition for treatment for the diseases which their specialties would cover? The only reason this question should ever come up in regard to the dentist is because, unfortunately, he is not usually willing or able to diagnose or prescribe for physical conditions outside of the oral cavity. The ophthalmologist, the aurist, and every other specialist, having laid the foundation of their professional skill in the studies of general medicine, and turned their attention afterward to the specialty chosen, are fitted to, and unhesitatingly do prescribe, when necessary to change the conditions of the system, so as to favor the success of the contemplated operation. It is just as important that the dentist should be able to do this as any other specialist.

The diseases of the teeth are influenced by the diseased conditions of the other organs, and it is patent to every intelligent operator that his success in dealing with the teeth and mouth, the diseases of the alveolus, mucous membrane and antral cavities, will depend largely upon whether he can bring the general system to the condition which will favor his success. Until there is a toning up and strengthening of the general system it is often worse than useless for a dentist to operate.

This is not all. There are signs of diseases which come under the dentist's eye more serious than merely a run-down system, and he should be able to recognize such peculiar conditions in a degree, and at least understand the relationship between such conditions and the disease in his peculiar domain, even if he prefer to refer the case to a regular practitioner or specialist in some other branch. The dentist should be able to refer the patient to the physician, giving a fair diagnosis of the case. If a physician received a patient from a dentist with only the message: "Something is the matter; find out what it is and bring about a better state," he would not have a very high opinion of that dentist's standing as a specialist in medicine.

All our medical teaching is based upon the idea that for every specialist a general knowledge of the whole system of medicine is necessary. If it is not necessary for the dentist, why should he study anatomy and physiology? If it is not necessary, why should he study general pathology? It is necessary, for if he does his whole duty in his profession, he must have this knowledge to enable him to recognize and treat such general conditions. And more than that, it is necessary for the treatment of such diseased conditions as are recognized as belonging to his own specialty. The course of an alveolar abscess or necrosis of the jaw is the same as abscess in any other of the soft tissues and necrosis of any other bone.

DR. JOHN S. MARSHALL said that when a dentist did not feel able to treat systemically when it was necessary, it was the fault of our former faulty system of teaching in the dental schools. Until recently it was generally considered that a dentist had no need of the knowledge of general medicine. No one would go with a diseased eye to one who was not a graduate of medicine, and the feeling about dentists should be, as it is about the oculist. The dentist should be as able to treat general disease as any other specialist. He would be sorry if he could not prescribe for any condition which would hinder the success of the treatment for which the patient had come to him. Our field is the mouth, not merely the teeth. We must be able to treat systemic conditions which we observe indications of, and if we are properly educated so as to be able to diagnose them, why should we not?

DR. VIDA A. LATHAM said she would like to ask the Section a question bearing on the subject of the paper, and also upon professional ethics or etiquette. The question is, may a dentist suggest to a physician the necessity for a general line of treatment for disease of the general system? Dr. Latham related the case of a patient of hers, a young man about seventeen years of age, who was a hard student. His teeth had been sound and apparently good until he had an acute attack of pneumonia, when it was found that in the course of about four months, either because of the disease or of the treatment, cavities had appeared in nearly every tooth. When examination was made, the young man was so weak that he fainted in the office. Dr. Latham called the attention of his mother to the state of his teeth as well as to his physical condition, and recommended that he be put in charge of his physician, as she thought there was a serious danger of a relapse into pneumonia. The physician declared that he was in need of no further treatment. The consequence was as she had foreseen, a dangerous relapse, which leaves him at present with a second severe attack of pneumonia. Dr. Latham desired the opinion of the members of the Section as to whether she had done more or less than she should in sending the patient back to the physician for treatment.

Dr. E. S. Talbot said that the whole question of the relations of the dentist to systemic medication and to the general practitioner was affected by the faulty system of dental education, which is entirely wrong. He spoke of a gentleman who held a position in a medical college and also in a dental college. The faculty of the medical college was pleased with him, but after the first year the faculty of the dental college declared that his lectures were too far advanced for the dental students; that they did not care to go so deep into things, and they were dissatisfied with him on that account.

The condition and the ambition of the dental student of to-day is little better and little higher than it was twenty-five years ago. He only cares, in most cases, for just enough general medical and physiologic knowledge to get for him the diploma, and when he has secured that he forgets all that is not, in his opinion, necessary for his success as a dentist.

The specialists in other departments do not feel so. They generally have practiced a few years at least before taking up a specialty, and are competent to treat any diseased condition which presents itself to them. The dentist, on the other hand, could not diagnose diseases of the general system, and not being a graduate in medicine would not dare to treat them. For himself, he said that his education was all the wrong end front, as he had practiced before attending any college, and afterward had taken a medical degree. The proper course would be the exact reverse of this. First the medical degree, next the dental, and afterward the practice. There are comparatively few of his patients that he does not prescribe for systemically. He has no sort of hesitation in considering himself a part and parcel of the medical profession, and in such a case as Dr. Latham reports he would go to the family physician and point out to him the condition of the young man and his need of treatment. He often has cases where he sees conditions which make it necessary to send the patient to a physician.

DR. EDGAR PALMER said that he thought when a patient whose physical condition was such as to make it impossible for the dental work needed to be done came to the office, the dentist should prescribe what will alleviate the condition. There is a long list of troubles we do prescribe for, in preparing the system for the surgical operations in the mouth; but of course there is a common sense limit, and we should not take upon ourselves the responsibilities of the general practitioner.

DR JOHN S. MARSHALL said Dr. Latham's question had not been fully answered yet. It was, had she the right to go to the physician and report what she had discovered? He though she not only had the right, but that it was a duty.

DR. GEORGE V. I. BROWN agreed with the ideas which had been advanced, but thought that the education of dentists is improving and rapidly, too. The dentist should know how to prescribe, but should be careful not to encroach on the field of the general practitioner, and so raise feelings of jealousy. More trouble comes to the physician's patients from not consulting the dentist than comes to the dentist's patients from not consulting the physician. If he had a patient who was not getting from his physician proper treatment, he would not hesitate to recommend a change of physicians. This would rarely be necessary, but sometimes it would.

DR. ANDERSON said he saw no reason why the dentist should not apply all the knowledge he had in any direction for the benefit of his patients. If, however, the dentist neglects his own specialty to learn to practice general medicine, he would lose opportunities for advancement in his own special line.

DR. W. H. CARSON said that to discuss the question asked by Dr. Latham was equivalent to admitting that we were not specialists in medicine. If we are members of the medical profession, then, of course, we should consult with the family physician.

Unfortunately, the majority of the dentists are not properly educated; they are not competent even to write a prescription. This should be changed; we should be physicians first and specialists afterward. Dentistry is looked upon by students as a more easily acquired profession than medicine, and those who are unable to acquire the latter feel that the former is within their reach, and thus it comes that the class of students at dental colleges are less highly prepared, educationally, and probably mentally, too. This should be changed; as much should be required of the dental student in the way of study as of the medical student, and the requirements for matriculation to the dental student should be as high.

Dr. Taft regretted that the mass of the profession did not appreciate the necessity of a good general education as a foundation for the professional education. The low standard of education required for entering the colleges is not so much the fault of these institutions as it is the fault of the profession. Almost every student is sent to the college either directly from the office of a preceptor or, at least, has taken the advice of some dentist as to the step. This is where our responsibility is. If an unfit man asks your advice about studying dentistry, persuade him not to study it. Most of the dentists have very little knowledge of general medicine, yet if they would they could get this knowledge, and they should take hold and master it, and they would do so if they could appreciate the necessity for it. On the other hand, the medical profession do not appreciate the diseases of the mouth. This is because they have so many things in their course of study that the diseases of the special organs are not taught thoroughly in the colleges.

---

Dr. Annie Felton Reynolds, of Boston, who read a paper before the Dental Congress in Chicago, was the first woman to be graduated from a New England Dental College. She was one of two women to speak before the Dental Congress.

